# A scoping review on the obstacles faced by beta thalassemia major patients in Pakistan- Matter of policy investment

**DOI:** 10.3934/publichealth.2024057

**Published:** 2024-11-12

**Authors:** Ali Hussain Ansari, Saqib Hussain Ansari, Mubarak Jabeen Salman, Muhammad Usman Hussain Ansari, Rawshan Jabeen

**Affiliations:** 1 Medical college, Aga Khan University Karachi, Pakistan; 2 Children's Hospital, Karachi, Pakistan; 3 Community health science, Aga Khan University Karachi, Pakistan

**Keywords:** beta thalassemia, challenges, healthcare, Pakistan, policy, safe blood transfusion

## Abstract

Beta-thalassemia major (β-TM) is a genetic disorder, prevalent especially in the Mediterranean region, Southeast Asia, and the Indian subcontinent. With improvements in management over the years, β-TM has transitioned from a fatal childhood disease to a chronic condition. However, in Pakistan, there is still a lack of a comprehensive national policy and strategic plan, which has resulted in a growing number of β-TM patients, placing a substantial burden on individuals and the national healthcare system. This scoping review is aimed to understand obstacles faced by β-TM patients in Pakistan. For this review, 26 unique articles were identified by using the PRISMA flow guidelines. PubMed and Google Scholar were used with the MESH term Beta-Thalassemia Major AND Pakistan, and the duration was set between 2012–2022. Then, the reviewers created a spreadsheet using Microsoft Excel to add in the data from the studies selected. Inductive and deductive approaches were used for thematic analysis. Additionally, we critically analyzed the current landscape of β-TM in Pakistan. The main challenges in β-TM care in Pakistan are suboptimal transfusion services and a poor complication management. Due to the need of chronic blood transfusions, transfusion-transmitted infection (TTI) incidence within this patient population is high. These largely include hepatitis C, hepatitis B, and the Human immunodeficiency virus (HIV). TTIs impact the quality of life of these patients and their overall survival. Furthermore, psychosocial morbidities are also prevalent in β-TM patients, with increased levels of hostility, anxiety, and depressive symptoms, thus emphasizing the critical need for sustained psychological support. Access to quality treatments is constrained, with notable disparities between public and private sector hospitals. Additionally, the financial burden on β-TM patients is considerable, which contributes to economic strain and more hardships on the already suffering families. The review concludes that the absence of a unified national policy exacerbates these challenges, which results in an escalating burden of β-TM nationwide. To address these issues, essential recommendations include the following: the implementation of a standardized protocol for β-TM care, the enhancement of access to quality care, the provision of iron chelation therapy, and safeguarding safe blood transfusion practices. Prevention programs, along with increased public awareness and education about β-TM and carrier screening, are pivotal. Collaborative efforts with international partners and drawing insights from successful strategies in countries with similar β-TM burdens can aid in mitigating the overall impact of β-TM in Pakistan and improving the quality of life of the affected individuals.

## 1. Introduction

Beta-thalassemia major (β-TM) is a genetic disorder that is more prevalent in developing countries. The population of the Mediterranean area, the Indian subcontinent, and Southeast Asia are the most affected [1],[2]. Due to advancements in treatments and prevention in Pakistan, β-TM has transitioned over the years from a fatal childhood disease to a chronic condition [3]. β-TM is a serious medical and psycho-social dilemma. These patients are largely dependent on regular blood transfusions with iron chelation therapy. The life expectancy of these patients is thought to be lower than the normal population, even though patients in developed countries have managed to survive up to their fifth decade of life [4],[5]. β-TM has been shown to be associated with several systemic diseases including growth retardation, a delay in sexual maturity, hormonal issues such as thyroid, parathyroid, and sex hormone deficiencies, diabetes, and cardiovascular disorders. The causes of these disorders are multifactorial, including chronic hypoxia and anemia, iron overload, decreased somatomedin activity, multiple endocrinopathies, poor socioeconomic status, and ethnic or racial factors [1]. The World Health Organization (WHO) has designated the prevention of β-TM in developing countries as a priority. Pakistan has a population of approximately 225 million people and a β-TM carrier frequency of more than 5%; therefore, over 9 million carriers are present nationwide [6]. Additionally, β-TM is the most common genetic disorder in Pakistan, with an estimated 40,000 children registered as transfusion-dependent and 5000–9000 children born annually with the condition [6]. The main purpose of this review is to highlight the importance of β-TM in Pakistan and to suggest recommendations to improve the living experience of β-TM patients.

## 2. Methods

The total searched articles were 270, including 170 articles from Google Scholar and 100 from PubMed databases, which were published over the last 10 years (2012–2022). This includes relevant articles, health system policies, and reports. Furthermore, we included 25 articles that were published in languages other than English, and the duplicates were removed.

### 2.1. Inclusion criteria

The studies included in this review discussed the obstacles faced by β-TM patients in Pakistan between the years 2012 and 2022.

### 2.2. Exclusion criteria

The search strategy excluded study designs such as investigations into obstacles faced by β-TM patients and those that addressed medical complications and either physical or psychological disabilities.

### 2.3. Medical search term

**Figure 1.**
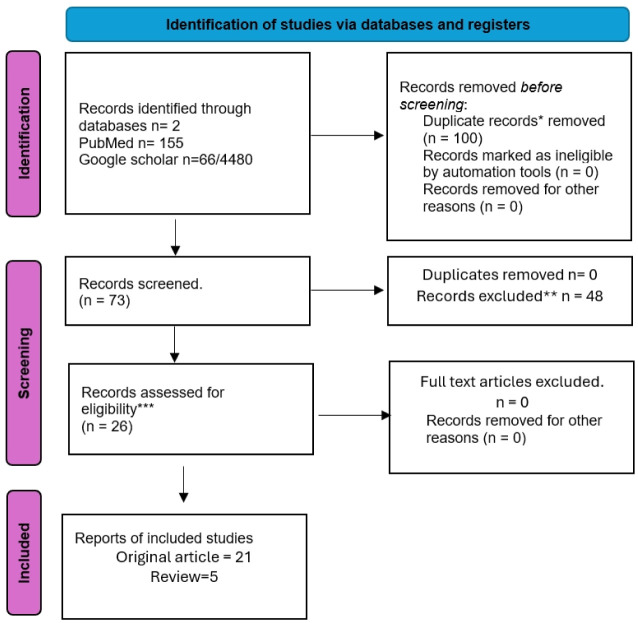
Prisma for systematic review: Study Flow Chart. (*Articles do not contain data in Pakistan; **Articles do not fall in eligibility criteria, irrelevant or low quality; ***Articles describes obstacles faced by β-TM patients in Pakistan.)

The following terms were used for the search: Beta-Thalassemia AND Pakistan during 2012–2022, which includes PubMed (155) and Google Scholar (4480). A total of 270 articles were scrutinized after a primary review by two different researchers, followed by an additional analysis by other members. Forty-eight articles were identified as unique, which were later reviewed; only 26 articles met the eligibility criteria (Figure 1).

### 2.4. Screening and selection of evidence source

The identified studies were stored in Endnote 20 (Table 1) and the duplicates were removed. Following this, two independent reviewers examined the titles of the identified studies using Microsoft Excel. Any disparities between the reviewers were resolved through discussion meetings, with the involvement of a third member of the research team if needed.

**Table 1.** Literature search.

S.NO	Title	Year of publication	Obstacles of Thalassemia major patients
1	A Systematic Review and Meta-Analysis on the Epidemiology of Hepatitis B and Hepatitis C Virus among Beta-Thalassemia Major Patients in Pakistan [7].	2021	All 33 articles yielded information on HCV prevalence, while 19 of them provided information on HBV prevalence. The overall sample size was 8554 that tested the prevalence of HCV in thalassemia patients. The sample size from the 19 studies that tested the prevalence of HBV was 6184. The overall pooled prevalence of HBV was computed to be 4.13%, while the pooled prevalence of HCV was 29.79%.
2	Thalassemia in Pakistan [8].	2022	No standard management protocols exist and blood transfusion remains the mainstay of management. Most of the population belongs to the lower socioeconomic strata, family units are large and therefore cannot afford to pay for treatment and management of their thalassemia child. Currently in Pakistan, at the national level, not a single thalassemia prevention program is available to counter this disease.
3	Efficacy and adverse effects of oral chelating therapy (deferasirox) in multi-transfused Pakistani children with β-thalassemia major [9].	2015	The most common adverse effects of oral chelating therapy (deferasirox) are abdominal pain, nausea, vomiting, and elevation of liver enzymes.
4	Assessment of Patients with β-Thalassemia Major, Undergoing Tertiary Care at a Regional Thalassemia Center in Pakistan [10].	2022	Secondary complications were assessed by physical examination of pallor, splenomegaly, ascites, and hepatomegaly.
5	Management of Thalassemia in Pakistan [11].	2016	However, very few centres furnish all these components of optimal thalassemia management under one roof. Management of Thalassemia in developing countries like Pakistan poses a major challenge. There are more than 40 Thalassemia centre's across the country. The majority of them are giving only transfusion support. The government does not provide the required support and the majority of the burden is borne by NGOs.
6	Epidemiology of Transfusion Transmitted Infection among Patients with *β*-Thalassaemia Major in Pakistan [12].	2016	Out of the 1253 multiple transfused patients, 317 (25.3%) were infected with TTIs. HCV was positive in 273 cases (21.7%), HBV in 38 cases (3.0%), and HIV in 6 cases (0.5%). Conclusion. HCV was the leading TTI in multi-transfused thalassemia major patients in the study. The presence of HIV in thalassemia patients is a recent disturbing development in Pakistan. Improved regulation of blood banks including the use of internationally or nationally evaluated kits will bring down the incidence of TTIs in transfusion-dependent *β*-thalassaemia patients.
7	Prevalence of HCV in β-thalassemia major patients visiting tertiary care hospitals in Lahore – Pakistan [13].	2014	Among 200 patients, 82 (41%) were found reactive for HCV antibody with an age range of 2 to 18 years with a mean age of 8.5 years. This study showed that haemodialysis patients and β-thalassemia sufferers were at higher risk of having HCV infection; the prevalence being 41%.
8	Prevalence of Transfusion Transmissible Infections in Beta-Thalassemia Major Patients in Pakistan: A Systematic Review [14].	2020	Among these, 166 studies met the inclusion criteria, and only 14 studies met the final criteria for qualitative synthesis. Analysis of 14 studies (n = 3786) showed the seroprevalence of hepatitis B virus (HBV) of 3.13% (0.66% to 7.4%) and hepatitis C virus (HCV) of 26% (5.56% to 68.2%). There were only two studies that reported HIV seroprevalence of 0% and 0.5% (n = 6). The rate of seropositivity for HBV and HCV was directly related to the number of transfusions, higher ferritin levels, and older age groups. There was an increase in the HCV rate with the increasing age of patients. Thalassemia patients, who were older than ten years of age, had an HCV rate of 22% compared to only 8.4% in patients younger than ten years of age. A comparison of HCV in healthy donors vs. thalassemia patients showed a rate of 1.9% vs. 13.1% for TM patients. The majority of the patients were males (51% to 88%). The seroprevalence of TTIs was higher in males than in females (73.4% vs. 26.6%). On average, a single TM patient is exposed to at least 17 different donors annually, requiring 1–2 transfusions every month. Our study highlights that the prevalence of transfusion-transmitted infections, especially HCV, is alarmingly higher (26%) in the TM population than in the general population. There is limited data regarding the prevalence of HIV, syphilis, and malaria in this population. This is mainly due to a fragmented system of blood transfusion, weak regulations, and lower rates of voluntary blood donations.
9	Quality of Life of Pakistani Children with β-Thalassemia Major [15].	2018	Age, income, education, pre-transfusion haemoglobin (Hb), serum ferritin level, pain, death due to β-thal, monetary issues, and pain were significantly associated with TranQoL scores. It was not only the clinical conditions but also life alterations, social relationships, and psychological events that also loomed in the improvement of treatment outcomes.
10	Endocrine complications in patients of beta thalassemia major in a tertiary care hospital in Pakistan [16].	2012	Our data revealed that a significantly small percentage of those under care received regular endocrine follow-up. Male hypo-gonadal abnormalities had the highest probability; 100% of the tested patients had decreased levels of testosterone, while 95.2% had raised serum creatinine levels. Parathyroid dysfunction was noted in 40% of the patients. Of those screened 29.4% had blood glucose levels in the diabetic range and 11.8% of the tested patients had reduced levels of FT4.
11	Status of Hepatitis B and C in Beta Thalassemia Major Patients [17].	2016	Among a total of 80 patients, 31 (38.7%) were hepatitis C positive and their mean number of blood transfusions received was 24 + 6.9 SD. Patients with an increased number of blood transfusions per year were found to be more HCV positive with p value of less than 0.05. In total 4 (5%) patients turned out to be hepatitis B surface antigen positive a.
12	Thalassemia Patients from Baluchistan in Pakistan Are Infected with Multiple Hepatitis B or C Virus Strains [18].	2021	Eleven (2.8%) were hepatitis B surface antigen positive, and 72 (18.3%) had anti-hepatitis C virus (HCV), two of which were infected with both viruses. Only 22% of the children had been reached by the program for universal hepatitis B virus (HBV) vaccination which started in 2004. Half (51%) of the HCV-infected had also been HBV-infected. The HBV- and HCV-infected patients were older and had received more blood transfusions than the uninfected patients (P < 0.001).
13	Frequency of Hypoparathyroidism in Children Presenting with Beta Thalassemia Major in Tertiary Care Center [19].	2020	We found the frequency of HPT among 18 (22.5%) cases. (5.45 ± 7.17) years was the mean age of beginning HPT. Patients who had HPT were mostly on single chelation therapy using deferiprone (DFP). There was a high prevalence of hypocalcaemia found in 43 (53.8%) cases. Among 43 cases of hypocalcaemia, 24 (55.8%) patients were asymptomatic, and 20 (44.2%) were symptomatic in which paraesthesia's & numbness were the most common complication.
14	Quality of life in patients with thalassemia major in a developing country [20].	2014	Parents of 36 (35.6%) of the children at times did not allow their children to play because of their disease. Twenty-eight (27.7%) stated difficulty in mingling with children of their age. Seventy-one (70.3%) of the patients reported that at some or all times they were worried about their future life and career while 70 (69.3%) admitted being taken extra care of by their friends and 56 (55.4%) by their teachers.
15	Establishment of a thalassemia major quality improvement collaborative in Pakistan [21].	2020	Approximately half (52%, n = 153) of the patients demonstrated severe myocardial iron overload (T2* < 10 Ms). The majority of the patients (58%, n = 175) were not on adequate chelation therapy. There was no difference in echocardiographic measures of the systolic and diastolic left ventricle among the different spectrums of iron-overloaded myocardium.
16	Social epidemiological analysis of risk factors and psychosocial burden of beta thalassemia major (bum) in Pakistan [22].	2021	The finding of the study revealed that socio-economic risk factors (lack of social and financial support, the sympathetic attitude of doctors and paramedics), cultural risk factors (religious restriction regarding prevention and screening of disease, with a strong emphasis on consanguinity), and disease-related factors (lack of provision of blood and medicines, and inadequate health system) are among the major risk factors associated with beta-thalassemia major.
17	Psychosocial Burden among Thalassemia Major Patients: An Exploratory Investigation of South Punjab, Pakistan [23].	2017	Among the total respondents, 50 (54.9%) responded that Thalassemia disease created an impact on their education. The majority [77 (84.6%)] of the respondents reported that they were not engaged in outdoor play equal to their friends. A greater number of the respondents [70 (76.9%)] were not satisfied with their body image and did not discuss their illness and its related problems with their friends. Majority of the respondents [71 (78.0%)] said that they felt different from their siblings. A greater number of the respondents [69 (75.8%)] said that they got the same attention from their parents. About 77 (84%) respondents said that their disease had a burden on their parents 55 (60.4%) of the respondents discussed that their disease limited their social life. Affected thalassemia major patients perceived a tremendous psychosocial disease burden.
18	Impact of Thalassemia on Quality of Life [24].	2016	Total 266 patients of 2–18 years of age and their parents were recruited. Male children and adolescents were 169 (63%) and 97 (37%) were female. Their mean age at diagnosis of beta thalassemia major was (10.43 ± 12.02) months. Children receiving one blood transfusion per month were 129 (49%) while 137 (51%) were receiving two or more than two blood transfusions per month. In general, Parents reported lower PedsQL 4.0 score as compare to their thalassemia children. Psychosocial health summary score was (75.37 ± 25.79) versus (70.73 ± 23.16) with p-value 0.04. Mean score for emotional functioning was (75.38 ± 28.89) versus (67.31 ± 23.51) (p = 0.00). No significant association of age, gender and number of blood transfusion was found on perception of health related quality of life.
19	Psychosocial Problems Faced by Thalassemia Major Patients of District Multan, Pakistan [25].	2018	Majority 106 (53%) of the respondents family monthly income was PKR Majority 106 (53%) of the respondents family monthly income was PKR < 20000 per month. Results indicated a significant association between the education level of thalassemia major patients and gender (X^2^ = 17.905a), (P = 0.000). Moreover, there was a significant association between the male and female thalassemia patients participation in extracurricular activities (X^2^ = 6.627a), (P = 0.007).
20	Are people getting quality thalassemia care in twin cities of Pakistan? A comparison with international standards [26].	2018	Results showed that almost half of the thalassemia patients (48.5%) were getting poor-quality of care. On average patients were getting only 63.93% of possible quality care for the disease. The most deficient quality area was the management of complications where patients were getting only 49.1% of possible care. Better quality of care was likely to be received by those patients who were educated, patients with educated fathers, those visiting private facilities, and those who were visiting facilities in Islamabad.
21	Perception of Parents of Thalassaemic Child to Thalassemia in Pakistan [27].	2021	Only 14% of participants reported that their affected children were receiving iron chelation adequately, while 38% received intravenous chelation only at the time of blood transfusions.
22	Transfusion-transmitted infections, its risk factors and impact on quality of life: An epidemiological study among β-thalassemia major children [28].	2022	Two-fifth (39.9%) of them were found to have TTIs with hepatitis C being the most common (34.5%), followed by hepatitis B (4.5%) and human immunodeficiency virus (1.8%). In the multivariable model, place of residence (adjusted odds ratio [AOR] – 2.23 [1.19–4.17]), per capita monthly family income (AOR – 1.84 [1.10–3.07]), and blood transfusion frequency (AOR – 1.19 [1.10–1.29]) were significant predictors of TTIs adjusted with their age, age at diagnosis, last pre transfusion hemoglobin level, size of spleen, and caregivers knowledge regarding the disease. The study participants with TTIs had a lower QoL compared to others as there were significant differences between the total QoL scores ([49.9 ± 15.6 vs. 57.4 ± 15.5], P ≤ 0.001) and its various domains.
24	Thalassemia in Pakistan: A Forward-looking Solution to a Serious Health Issue [29].	2020	Blood safety has particular relevance in Pakistan due to the high prevalence of transfusion transmissible infections, mainly hepatitis B and C, and in some cases, even HIV/AIDS, in chronic transfusion recipients such as thalassemia [7]. The main culprit in this regard is the common use of rapid manual screening kits that are not properly evaluated and validated. In general, these kits have poor sensitivity and are unable to detect low- or medium-grade infections and thus show false-negative results [8]. As a result, unsafe blood transfusions are an important driver of the hepatitis epidemic in the country. The purchase and validation of blood screening kits need to be properly regulated by the government. One of the common side effects of frequent blood transfusions is the development of alloantibodies in thalassemia patients, which destroy the transfused red blood cells unless they are perfectly matched [9]. This causes a reduction in the intervals between subsequent transfusions and other complications in addition to misery, further risk of acquiring infection, and expense. This hazard can be prevented by the routine use of leukocyte filters during blood transfusions as recommended for all patients, especially for chronic transfusion recipients [10]. However, the additional cost involved is a barrier to the routine use of these leukocyte filters. The market cost of a single-use leukocyte filter is approximately 10 US dollars. About half of this market sale cost is the customs duty (personal communication). If the government could waive off this customs duty, the leukocyte filters could become more affordable, and hence, improve the quality of treatment of thalassemia patients.
25	Quality of life among beta-thalassaemic major children presenting at Federal Government Hospital Islamabad, Pakistan [30].	2022	Of the 87 subjects, 47 (54%) were males and 40 (46%) were females. The overall mean age was (10.71 ± 1.99) years. The mean quality of the scale score was (50.24 ± 18.88). Poor quality of life was found among 33 (37.9%) children. The quality of life had a significant association with age 7–9 years, male gender, and blood transfusion frequency 2 or more (p < 0.05). The adjusted odds were also significant with age and blood transfusion frequency (p < 0.05). The overall mean score was significantly related within age groups and frequency of blood transfusion (p < 0.05), whereas physical and emotional domains were significant with age (p < 0.05), while the four domains of physical, psychological, social, and educational were associated with frequency of blood transfusion (p < 0.05).
26	Out Of Pocket Expenditure on Thalassemia Major and Its Implications on The Household Economics [31].	2022	Treatment expense of entitled patients from FFH hospital (a public hospital that offers entitlement to the families of retired army personnel) was compared with that of non-entitled patients coming to PIMS (a public sector general hospital). The total expense incurred on treatment by the end of the month was PKR 5000–10,000 (USD31–62) in FFH, while at PIMS, the total expense incurred on treatment by the end of the month was around PKR 80,000 (USD500). Around 37% of families having an average monthly income of PKR- 25,000Rupees (USD150) only, sold their livelihoods, 31% compromised on their children's education expenses and 23% percent curtailed the health expenses of the other children.

### 2.5. Data extraction

Specifically for this review, the reviewer created a spreadsheet using Microsoft Excel based on the data taken from the studies. To ensure they included pertinent information, two staff members went over the data extraction table studies. Every study was examined for pertinent data, including the study results, information about the challenges associated with β-TM, and studies that addressed medical issues, medical conditions, and mental health conditions.

## 3. Results

### 3.1. Transfusion-Transmitted Infections and other adverse effects relating to management

Transfusion-transmitted infections (TTIs) are a significant concern for β-TM patients, as they are at a higher risk of acquiring these infections due to frequent blood transfusions. The most common TTIs among β-TM patients in Pakistan include; hepatitis C, hepatitis B, and the human immunodeficiency virus (HIV) [7]. According to a study, of the 1253 patients who were transfused multiple times, 273 (21.7%) were found to have an HCV infection, 38 cases (3.0%) had an HBV infection, and 6 (0.5%) had an HIV infection [13],[17]. Patients who received blood transfusions that are more frequent had low pre-transfusion haemoglobin levels and large spleen sizes, which increased their risk of acquiring TTIs and iron chelation [9],[10],[14],[21]. The treatments required for β-TM patients to maintain vitality can also increase the risk of TTIs. Moreover, it was seen that the majority of the patients (58%, n = 175) were not on an adequate chelation therapy [21]. Similarly, another study suggested only 14% of the patients were able to adequately receive iron chelation, while 38% received intravenous chelation only at the time of blood transfusions [27]. Transfusions can cause hypocalcaemia, complications of which are paraesthesia and numbness, and account for 44.2% of symptomatic cases and 55.8% of asymptomatic cases [19],[28],[32]. Moreover, the impact of TTIs on the quality of life (QoL) of β-TM patients is a concern [28],[32]. Additionally, our results showed that 22.5% of cases had hyperparathyroidism, with a mean age of (5.45 ± 7.17) years at the onset. There were many male hypogonadal abnormalities: 100% had low testosterone levels, 40% had parathyroid dysfunction, 29.4% had blood glucose levels within the diabetic range, 11.8% had decreased FT4 levels [16],[28], and a significantly lower proportion of patients receiving care had regular endocrine follow-ups. A study in Lahore reported the quality of life of β-TM patients on the PedsQL 4.0 scale, with an average score was (68.58 ± 26.9), which indicates a moderate impact on the patients' well-being [24].

### 3.2. Psycho-social issues

A psychosocial morbidity in β-TM patients was seen, which revealed higher levels of hostility, anxiety, obsessive symptoms, somatic symptoms, and depressive symptoms. The QoL was adversely affected, particularly in the social and psychological domains. More than half of the participants (54.9%) reported an impact on their schooling due to β-TM. A significant percentage (84.6%) indicated reduced outdoor play, and a considerable portion (76.9%) expressed dissatisfactions with their body images, and refrained from discussing their illness with friends. While a sizable group (75.8%) believed they received equal attention from parents, the majority (78.0%) felt different from their siblings. A substantial majority (84%) acknowledged the disease's effect on their parents, and a significant proportion (60.4%) felt that their disease limited their social life. This underscores the notable psychosocial burdens faced by individuals with β-TM [33]. Moreover, a lower maternal education was seen to be associated with a higher prevalence of psychopathological tendencies. In a study on β-TM children at a federal government hospital in Islamabad, Pakistan, 37.9% of the children were found to have a poor QoL. The study found significant associations between the QoL and age (7–9 years), the male gender, and blood transfusion history [30]. Clinical and psycho-social experts should team up to provide an ideal consideration to their patients. Patients with β-TM may lack self-confidence due to the fear and anxiety caused by the chronic nature of the disease, as well as physical changes and complications [23],[25]. The majority of the respondents (53%) reported a monthly family income below PKR 20,000 (USD71), which added to their stress. A study revealed a significant association between the education level of β-TM patients and gender (X^2^ = 17.905a, P = 0.000). Additionally, a significant association was found between the participation in extracurricular activities among male and female β-TM patients (X^2^ = 6.627a, P = 0.007) [25]. The research indicated that a significant psychosocial burden was experienced by β-TM patients, as evidenced by the fact that 84% of the respondents said their illness burdened their parents and 60.4% talked about how it limited their social life [23].

### 3.3. Limited availability of quality treatment

Patients faced challenges in accessing quality treatments due to the absence of a cohesive national policy and strategic plan, leading to an increasing patient number and an increased strain on the healthcare system [12]. A study in Islamabad and Rawalpindi revealed that even though management for β-TM is available in both private and public hospitals, the quality of care is suboptimal, especially in managing complications [26]. Despite being a member of the Thalassemia International Federation, Pakistan has not yet implemented international-level protocols for standardized care [26],[34]. Efforts have been made to define the criteria for good quality care by constructing management guidelines for the disease; however, the implementation of these guidelines in Pakistan is limited [26]. The Punjab β-TM Prevention Project (PTPP) is a government-funded provincial intervention aimed at reducing the incidence of β-TM in Pakistan. The project includes a cascade screening for biological relatives of β-TM patients; however, the overall quality of care for β-TM patients in the country is still sub-partial [35]. A study in Rawalpindi and Islamabad found that β-TM patients received low quality care, with large gaps in staff training, a lack of diagnostic equipment and kits, and an inadequate institutional coordination, particularly in public hospitals [36]. Despite the establishment of multiple private and NGO-run thalassemia facilities around the country, the service quality greatly varies. While some clinics provide optimal care, others lack the requisite technical knowledge, and frequently solely focus on blood transfusions while ignoring critical therapies such as iron chelation therapy and other needed medical support [17],[29].

### 3.4. Financial burden

The financial burden on β-TM patients in Pakistan is substantial, with significant out-of-pocket expenditure for the management of the disease [37]. The high cost of treatment and management contributes to a catastrophic level of healthcare expenditure for many families, thus leading to financial strain and hardships [35],[38]. A study from Abbottabad showed that the monthly expense in management of β-TM in public sector hospitals is around PKR 5000–10,000 ($18–36), as compared to the private sector, where it is approximately PKR 80,000/$287 a month. Approximately 37% of the families in the study had a monthly income of around PKR 25,000/$89 [37], which put a great deal of monetary pressure on the families, and compelled them to borrow money and mortgage their property.

### 3.5. Lack of policy implications

In the absence of a coherent national policy and strategic plan, the number of β-TM patients in Pakistan is believed to be increasing, though the exact burden of the disease remains unknown [36],[39]. As a result, despite being a preventable blood disorder, β-TM in Pakistan continues to increase in number and is a cause of misery to the patients and their families [14]. A significant proportion of the blood transfusions which are performed are for β-TM patients. There is a need to better address this situation, which is a burden on the national healthcare and blood transfusion systems in Pakistan.

### 3.6. Landscape in the context of Pakistan

β-TM requires quality care and treatments to increase a patient's life expectancy. This can be seen in developed countries; however, Pakistan is still way behind [35]. Pakistan is a developing country, with a population estimated at more than 200 million. Limited resources and the high prevalence of non-communicable diseases are the main burdens on the healthcare system. The WHO has initiated collaborative institutes that work on genomic techniques for public health. These centers were launched in 22 countries, 36% of which are developing nations [40]. β-TM is not randomly distributed in Pakistan: families with more abundant intermarriages are at a higher risk due to gene frame-up and gene proliferation. Therefore, to enhance the life expectancy of β-TM patients, mass screening is not considered cost-effective nor an effective strategy for controlling this disease in Pakistan [35]. β-TM treatment is available in both public and private sector hospitals in Pakistan; however, the quality of care provided is below international standards, and there is an urgent need to improve the quality of care for β-TM patients in the country [26]. Additionally, the legislation does not have a mechanism for the implementation of treatment protocols, which receives a large pushback due to a lack of finances and human resources. In Pakistan, blood safety is a significant concern due to the high prevalence of TTIs, mainly hepatitis B and C. With an increasing number of new β-TM patients' day by day, the prevalence is on the rise, and efforts are being made to improve the treatment plan and quality of life of this large patient population. But for now, these improvements are limited to private sector hospitals, which charge a large sum for their services [4],[41].

The public authority of Punjab (the biggest province of Pakistan) launched the Punjab Thalassemia Prevention Program in 2012, which provides prenatal testing to couples with an already present child with β-TM, as well as screening for their relatives. Moreover, people who want to know about their thalassemia carrier status can volunteer under this program. Moreover, genetic counselling is provided to at-risk couples about prenatal screening and conceptive alternatives [42]. The burden of β-TM in Pakistan is significant, and several strategies can be implemented to improve the situation. Some suggestions include implementing the standard protocols from the Thalassemia International Federation and putting in efforts to improve the quality of life of these patients. Complication screening and management was found to be the most deficient area in the quality of β-TM care, which should be immediately dealt with [43]. Most importantly, prevention programs, including extended family screening and chorionic villus sampling for prenatal diagnosis, can help to decrease the incidence of β-TM.

### 3.7. Impact of COVID-19 pandemic on β-TM

In Pakistan, the COVID-19 pandemic has had a major effect on β-TM patient care. Studies revealed a significant decline in blood transfusions [44], with the median number of red cell transfusions per patient falling from four units to just one unit [45]. During the early stages of the pandemic, approximately 80% of thalassemia patients were unable to receive any healthcare services due to the severe disruption in access to healthcare services [46]. This was mostly brought on by lockdowns, which limited movement and the patients' fears of acquiring COVID-19 and kept them from receiving vital blood transfusions [44]. Additionally, the patients experienced negative effects on their mental health and general well-being. The patients' elevated levels of anxiousness, depression, and stress were found in a qualitative investigation, which highlighted the psychological toll that the outbreak had taken on this susceptible group [34].

## 4. Discussion

This review highlighted the current status and the landscape of obstacles which were reported in 26 articles published between 2012–2022. Additionally, the result revealed the impact of the COVID-19 pandemic on β-TM treatment in Pakistan. The major obstacles found were TTIs and other adverse effects which related to management, psycho-social issues, the limited availability of quality treatments, financial burden, and a lack of policy implications. A holistic approach should be taken, which emphasizes the psycho-social morbidity and other medical complications associated with β-TM. The out-of-pocket expenditure on healthcare should be discouraged, and authorities should work towards a system where high-quality healthcare is available to all, which decreases the burden on those who cannot afford it. National and international cooperation should be encouraged, taking cues from successful models such as Iran's eradication efforts. Pakistan can endeavor to mitigate the impact of β-TM and to improve the well-being of those impacted by the disease by putting these tactics into practice. In higher income countries such as Qatar, a study reported that adolescents with β-TM exhibited considerably lower and more variable overall QOL scores (69.1 ± 16.8) than healthy children (77.5 ± 2.8) (p < 0.001). β-TM individuals reported a reduced school functioning (38.8 ± 22.7) compared to the healthy controls (71.2 ± 15.2) (p < 0.001) [47]. Another study reported that the main factors which contributed to the high prevalence of β-TM were misinformation about the condition, stigma, and marriage dissolutions brought on by the illness. Additionally, superstitions and incorrect interpretations of religion, along with the ensuing practices, were important predictors of the psychosocial burden of β-TM among non-consanguineous and consanguineous couples [48].

Initiatives should be taken towards β-TM education and public awareness, along with support for prenatal diagnoses, carrier screenings, and genetic counselling, all of which can produce a large difference in the prevention of the disease. Furthermore, prenatal screening is available at a limited number of centers, though it is expensive; in order to utilize these services more effectively, prenatal diagnostic screening should be made free of cost. Moreover, to mitigate the fear of undergoing prenatal tests, a clear explanation with reasoning alongside the strong advocacy for its importance is required for its acceptability in a Muslim country such as Pakistan [49]. A drastic change is needed in the blood banks of Pakistan: the National Blood Transfusion Authority should regularly evaluate them to make sure that safe transfusion practices are being performed to decrease the rates of TTIs.

Recent advances in genome editing technology, particularly the introduction of base editors (BEs), are expanding the possibility for curative medicines in Pakistan [50]. Traditional genome editing techniques, such as CRI-SPR-Cas9, rely on double-strand breaks (DSBs), which can lead to unexpected consequences such as significant insertions or deletions, chromosomal rearrangements, or off-target effects [51]. Z. Abbas et al. found that BEs provided a more precise alternative by converting one DNA base pair to another without producing DSBs, and hence decreased these dangers [20]. T Arif et al. noted that genome editing technologies now enable precise adjustments to genomic sequences in eukaryotic cells, potentially providing therapies for a variety of hereditary illnesses [49]. Aga Khan University (AKU) researchers are pioneering the development of a revolutionary gene editing therapy based on CRISPR/Cas9 [20]. This novel therapy could be provided as a medication to fix defected genes, such as the hemoglobin subunit beta (HBB) gene, which is known to be defected in β-TM and sickle cell disease. By “snipping” and fixing the defective gene, this technique aims to cure many severe illnesses, which represents a huge development in Pakistani medical treatments.

The understanding and treatment of β-TM have greatly advanced in Pakistan due to the active involvement of several worldwide networks in the condition's study and management through campaigning, research, and education. The Thalassaemia International Federation (TIF) is a global organization devoted to preventing and controlling thalassemia. TIF offers crucial guidelines for diagnoses and treatments and has an extensive database of thalassemia cases [21]. The Human Variome/Global Variome Project (HVP) has launched the Global Globin Network (GGN), which focuses on hemoglobinopathies [51]. The objective is to enhance β-TM diagnoses and management through international collaborations by building a capacity for genomic diagnostics, clinical care, and research in low- and middle-income countries [20]. In an effort to gain a better understanding of the genetic variables that determine the severity of hemoglobinopathies, such as β-TM, the International Hemoglobinopathy Research Network (INHERENT) investigates the function of genetic modifiers in these disorders [43]. These global networks play a critical role in improving clinical procedures, expanding research, and eventually improving the lives of Pakistani individuals suffering with β-TM.

This scoping review has focused on obstacles of thalassemia major patients; however, parents and system related issues also have their magnitude. Thus, further reviews need to explore more information.

## 5. Conclusions

In conclusion, a comprehensive strategy is needed to address the issues related to β-TM in Pakistan. It is essential to provide safe blood transfusions, to improve iron chelation therapy, and to increase access to high-quality healthcare. The sobering fact that the average yearly cost of treating a child with β-TM surpasses the average annual income highlights the pressing need for an increase in access to healthcare.

## Use of AI tools declaration

The authors declare they have not used Artificial Intelligence (AI) tools in the creation of this article.
